# Advanced Experimental Technology, Theory and Numerical Methods in Geomaterials and Concrete Materials

**DOI:** 10.3390/ma19071464

**Published:** 2026-04-05

**Authors:** Wenhui Sun, Yifei Li, Runyu Liu, Shuyang Yu

**Affiliations:** 1 School of Transportation and Civil Engineering, Nantong University, Nantong 226019, China; 2533310008@stmail.ntu.edu.cn; 2 Department of Mechanical Engineering, Huzhou University, Huzhou 313002, China; 18552780325@163.com

Geotechnical and concrete materials are used as core carriers for infrastructure construction. Their mechanical properties, durability, and environmental adaptability directly determine the safe service life of engineering structures [1,2,3,4,5]. In situ testing and microscopic characterization techniques are employed in terms of experimental techniques, enabling the accurate capture of these materials’ multi-scale properties. At the theoretical research level, multi-field coupled constitutive models and micromechanics theory are applied, providing an integral framework for the prediction of material properties. In the field of numerical methods, multi-scale simulation is adopted, significantly improving the efficiency and accuracy of engineering problem solutions.

Experiments serve as the basis for revealing the intrinsic material characteristics [6,7]. Full-scale coverage—from macroscopic mechanical responses to microscopic structural evolution—has been enabled by current technologies. Choi et al. [8] investigated the natural and artificial aging effects on the deformation behaviors of Al–Mg–Zn alloy sheets, while Wang et al. [9] optimized thixotropic slurry ratios through drag reduction effect tests for pipe-jacking construction in pebble stratums. The main progress is reflected in the following two aspects: (1) In situ testing technology: Traditional laboratory sample testing cannot easily replicate the complex engineering site environment; therefore, for in situ testing technology [10], testing equipment is directly deployed in engineering scenarios, which enables real-time monitoring of material properties. For example, fiber optic sensing in situ testing systems are used during tunnel excavation. Thereby, continuous monitoring of stress and strain distribution in surrounding rock is enabled, with a resolution of 1με, effectively capturing its time-dependent deformation after excavation and unloading. For concrete structures, embedded piezoelectric sensors are applied, achieving real-time monitoring of internal crack initiation and propagation, as well as providing early warning of structural durability failure risks. The advantage of such technologies lies in eliminating the “laboratory-site” performance deviation. Real parameters are provided for engineering design. (2) Microscopic characterization technology: The deterioration of macroscopic material properties originates from the evolution of microstructures. The application of technologies such as scanning electron microscopy (SEM) [11,12,13], X-ray diffraction (XRD) [14,15,16], and nuclear magnetic resonance (NMR) [17,18,19] has enabled quantitative analysis of microstructures and compositions. For example, SEM–energy dispersive spectroscopy (EDS) combined technology is utilized to observe concrete after freeze–thaw cycles. It was observed that ice crystal growth increases the porosity of the interfacial transition zone (ITZ) from 8% to 15%, thereby resulting in a reduction in macroscopic strength. Low-field NMR technology [20] is employed to characterize the pore water distribution of saline soil, confirming that salt crystallization clogs capillary pores and reduces soil permeability. Moreover, an atomic force microscope (AFM) can be used to observe the material nanoscale surface morphology, providing direct evidence for understanding the mechanical properties of cement hydration products such as calcium-silicate-hydrate (C-S-H) gel. It is found that the elastic modulus of C-S-H gel increases from 10 GPa to 30 GPa with increasing density; thereby, this relationship lays an experimental foundation for mesoscopic theoretical modeling.

Concrete, as a multiphase composite material, has a microstructure consisting of the following components: mortar, aggregates, interfacial transition zone (ITZ), and pores. Among these components, mortar is composed of cement hydration products and unhydrated particles. It wraps around aggregates, hardens to form a matrix, and transfers stress. Unlike homogeneous materials, the fracture process of concrete exhibits significant multi-scale heterogeneity characteristics, ranging from the fracture of cement hydration products such as calcium-silicate-hydrate (C-S-H) [21] at the microscopic scale, to aggregate–matrix interfacial transition zone (ITZ) debonding [22] at the mesoscopic scale, and finally to the macro-crack propagation at the macroscopic scale. The multi-scale defect evolution and coupling ultimately determine its fracture mode and the law of bearing capacity degradation.

Micromechanics starts with the internal structure of materials, such as aggregates, pores, and interfaces, establishing the correlation between microscopic parameters [7,23] and macroscopic properties. Regarding modeling, Zhang et al. [24] proposed a concrete mesostructure modeling method via random radius fields and rigid body dynamics packing. Moreover, Zhu et al. [25] and Li et al. [26] utilized Discrete Element Method (DEM)-based mesoscopic modeling to simulate cracking processes in concrete under tensile stress and in fissured specimens, respectively, thus providing a basis for optimal material design. Regarding concrete, the random aggregate mesoscopic models [27,28] reveal the intrinsic mechanism by simulating the spatial distribution of aggregates, mortar, and the interfacial transition zone (ITZ), determining that an increase in the aggregate volume fraction ranging from 60% to 70% increases the compressive strength of concrete by 15% as well. This mechanism is attributed to ITZ’s reduced area ratio, which decreases the stress concentration effect. Regarding geotechnical materials, the particle flow micromechanical theory explains the dilatancy phenomenon during sand shearing by simulating the contact forces and movements between particles via the discrete element method (DEM) [29]. This phenomenon originates from the change in pore volume caused by particle rearrangement. Additionally, Umar et al. [30] developed a novel hybrid ensemble learning approach for precise joint roughness coefficient prediction, while Ziccarelli [31] explored the mix design of pervious concrete for geotechnical applications.

Geotechnical and concrete materials are often subjected to multi-field engineering actions of temperature, moisture, mechanics, and chemistry [32]; however, traditional single-field constitutive models have not been able to meet the requirements. In recent years, multi-field coupled constitutive models based on continuum mechanics have been widely developed [33,34,35]. For example, thermal damage variables are introduced into the model for rocks in high-temperature environments. The attenuation of rock elastic modulus and the aggravation of plastic deformation caused by temperature rises are described via the “stress–strain-temperature” coupling equation. For example, within the framework of the Lee–Fenves model, Guan et al. [36] proposed a simplified plastic damage model for analyzing deep cut-off walls, adopting a fracture-energy-based tension–compression constitutive relationship to characterize their mechanical response. Damage factors are introduced based on the Sidoroff energy equivalence principle, which considers the different evolution processes of tension and compression damage of cut-off walls. For concrete under chloride ion attack, the model combines chemical reaction kinetics and mechanics theory. A quantitative relationship between the chloride ion concentration and concrete compressive strength is established, where the strength loss rate is predicted to reach 20%~30% within a 50-year service life. The key to such models lies in the reasonable definition of coupled variables, such as damage and chemical ion concentrations. For example, in the rock mechanics analysis of a nuclear waste repository, the multi-field coupled model successfully predicts the thermal cracking range of surrounding rock caused by high-level radioactive waste heat release. Theoretical support is provided for the repository design.

This Special Issue clarifies the multi-scale performance, microstructural evolution, and multi-field response mechanisms of geotechnical and concrete materials by integrating experimental techniques, theoretical modeling, and numerical simulations, providing support for infrastructure safety and material optimization. Future research could focus on refining coupled variables (e.g., dynamic damage under cyclic loads) and fusing multi-techniques to improve performance prediction precision, while also expanding studies on material behavior under extreme environments, ultimately advancing the long-term safe service and intelligent infrastructure design.
